# The Red Cross in Ireland

**Published:** 1919-07-26

**Authors:** 


### THE RED CROSS IN IRELAND.

### Something Wrong Somewhere.

In no part of His Majesty's dominions, strange as it may appear, has the work of the Red Cross been performed with greater enthusiasm and success than in Ireland. To the initiated this is not a phenomenon, but a natural consequence of the antagonism of a section of the people to British aims and ideals. The other section, with patriotic impulse, shouldered the burden, and resolved to do by itself what ought to be shared by the whole population.

### A Constitutional Weakness.

In the early days of the war here, as elsewhere, inherent weakness of the British Red Cross organisation was keenly felt. I refer to the fact that the necessary legal licence provided by the Geneva Convention, and granted by the War Departments of belligerent countries with the object of enlisting civilian effort, was throughout this Empire conferred on two Associations, the British Red Cross Society and the Order of St. John. It sometimes happens that rivalry is a factor for good. It is pointed out by economists, for example, that railway, steamship, insurance, and similar companies, duplicated or quadruplicated, provide the public with better and cheaper service than would obtain under a system of State control and monopoly. This result, unfortunately, did not accompany the dual control of the British Red Cross. This is demonstrated by the fact that a much larger proportion of the peoples of America, France, Germany, and Japan enrolled as Red Cross workers than in the United Kingdom and in the Dominions. In America everybody who had a dollar paid it, and became a soldier of the Red Cross army. Here there was always the *arrière pensée*—what is to happen when the war is over? Will A or B remain a member of the B.R.C.S., or of the St. John Ambulance Brigade? And, even after the two were consolidated for the period of the war under a Joint Committee, although with admirable patriotism rivalry was driven under, the fact that rival interests existed was always apparent, and there is no room for doubt that this is mainly responsible for preventing the carrying out of a campaign of thoroughness which would have resulted in a complete or an almost complete national enrolment. This weakness might possibly have been provided against at the outset had the colossal extent and the long duration of the conflict been foreseen. As it was, the best was made of an imperfect machine, and now that the war is over British Red Cross workers of both Societies look back with pleasurable pride on their almost miraculous achievement.

### A Kink in the Machine.

The object of these remarks is to call attention to what the writer regards as a kink in the machinery, which, if not rectified, must seriously interfere with its future efficiency. This is very plainly visible in Ireland, and in all probability also operates in England. During recent weeks the Order of St. John issued a direction that its male members should abandon khaki, and substitute in its stead black raiment. A second order prohibited its members as such, male and female, from participating in Peace processions. I am unable to explain the motive which prompted these orders. But I can state, without fear of contradiction, that the St. John Ambulance Brigade in Ireland owes its present very substantial strength to the men and women who joined its membership as Red Cross workers, and the great majority of these belong to a class who find the purchase of any kind of clothing just now a difficult problem. Many men are getting their old clothes turned rather than pay the price of a new suit. When they took up Red Cross service in Dublin they obtained khaki uniforms and wore them with pride. Eight hundred men spent their spare hours in stretcher drilling and learning first aid in order to have the privilege of skilfully unloading hospital ships which disembarked at this port, and in carrying the wounded to hospital. They travelled in hospital trains to Belfast and Cork, and, in addition to this, they assembled in their scores or fifties, when requested, at Red Cross fêtes and functions, and with delight performed menial offices, knowing that they were "helping to win the war," and paying a tribute of service to our gallant men-at-arms. Irish work in connection with hospital ships was, indeed, superb. The R.A.M.C. handed over their task to these Red Cross men, absolutely and altogether. From the "cot" in the ship the wounded were transferred to the Red Cross ambulance, and, with the co-operation of Red Cross automobiles, taken right up to the hospital beds by Red Cross men in black, in khaki, and in the blue uniform of the B.R.C.S. The St. John khaki men are now ordered to change their clothes, and with one accord they exclaim—"We are scrapped!"

The last straw, however, is the order to men and women alike, not to march in Peace processions, but, instead, to wear brassards and do duty, should accidents occur, in the crowds. This, it is pointed out, is the legitimate and proper work of the Brigade. Just think of it!

### The Red Cross Licence.

I venture to suggest that in issuing this Order the hierarchy of St. John forgot that, in addition to its fundamental mission, the Order is a licensed civilian Red Cross agency. Prior to the war its work in Ireland was comparatively obscure. From a humanitarian point of view it was, no doubt, excellent—a fact which none will deny. But in numbers and in influence it was a weak and struggling institution. The war brought to it an enormous accession of strength, simply because men and women joined in hundreds for Red Cross work. On this account an order forbidding participation in the procession of Peace appears not only arbitrary, but altogether unjustifiable. To move about amongst crowds of sightseers wearing brassards and in readiness for emergencies may be, and doubtless is, St. John Brigade work. It is certainly not Red Cross work. And I protest that the Red Cross, the child of war, is intimately associated with Peace rejoicings. Let us get down to first principles. The real Red Cross, if I may use the term, is the R.A.M.C. and its naval counterpart. Civilian effort, although of priceless value to every belligerent country, is only tagged on. This is obvious from a superficial perusal of the provisions of the Geneva Convention.

### A Slap in the Face.

I repeat that I am unable to suggest what was the underlying motive of the orders referred to. I can only testify that in Ireland they have given offence which will inevitably be followed by demobilisation. St. John Red Cross men and women feel that they have received a slap in the face, especially in view of the fact that in a Dublin crowd there is no necessity whatever for ambulance booths. A single detachment would suffice in the most exceptional cases. If a national or international call is made, I think it is plain that in this field something will have to be done by way of reconstruction. IERNE.

